# Exploring the impact of a caregiver education program within the Comprehensive Resilience‐Building Psychosocial Intervention (CREST) for people with dementia: informal caregivers' perspectives

**DOI:** 10.1002/alz.087307

**Published:** 2025-01-09

**Authors:** Dympna Casey, Siobhán Smyth, Priscilla Doyle, Niamh Gallagher, Barbara Whelan

**Affiliations:** ^1^ University of Galway, Galway Ireland

## Abstract

**Background:**

As part of the pilot Comprehensive REsilience‐building psychoSocial intervenTion (CREST) for people with mild to moderate dementia living in the community, a carer education program was developed. The aim of the program was to develop carers’ knowledge and skills regarding dementia to enable them to respond more confidently to the needs of the person with dementia, provide them with ‘me time’, with an opportunity to focus on their own health needs, meet other carers, and share experiences. This study explores the carers’ experience of the program, which consisted of six weekly, 2‐hour sessions with each week covering a different topic.

**Method:**

Qualitative data were collected from group/individual interviews with the carers during the education programme and at the end of the CREST intervention. Attendance rates were recorded and evaluation forms completed by carers at the end of each session. Qualitative data were analysed using thematic analysis and descriptive statistics were used for quantitative data.

**Result:**

Nine carers completed the intervention with an average of 91% attendance (range: 67‐100%). Evaluations were very positive with all carers agreeing that the content of each session was helpful, relevant to their needs, and met their expectations. A key motivator to participate was to gain more knowledge and understanding about dementia and learning communication strategies were particularly useful. The support and learning that carers got from other group members, the social interaction and the emphasis on self‐care were important elements of the programme.

**Conclusion:**

Carers of people with dementia need good support to enable them to manage the challenges of caregiving. The CREST intervention’s carer education program played a crucial role in assisting the carers in managing stress through better communication strategies. It also facilitated the creation of a supportive network among the carers and encouraged them to prioritise their own needs, ultimately enhancing their ability to fulfil their caregiving role.

